# Comparison of neostigmine and sugammadex for hemodynamic parameters in neurointerventional anesthesia

**DOI:** 10.3389/fneur.2023.1045847

**Published:** 2023-04-17

**Authors:** Yu-Hsun Tsai, Chun-Yu Chen, Ho-Fai Wong, An-Hsun Chou

**Affiliations:** ^1^Department of Anesthesiology, Chang Gung Memorial Hospital, Linkou Medical Center, Chang Gung University, Taoyuan City, Taiwan; ^2^Department of Diagnostic Radiology, Chang Gung Memorial Hospital, College of Medicine and School of Medical Technology, Chang-Gung University, Linkou, Taiwan

**Keywords:** intervention, sugammadex, general anesthesia, hemodynamic, cardiovascular—history

## Abstract

**Introduction:**

Hemodynamic stability is important during neurointerventional procedures. However, ICP or blood pressure may increase due to endotracheal extubation. The aim of this study was to compare the hemodynamic effects of sugammadex and neostigmine with atropine in neurointerventional procedures during emergence from anesthesia.

**Methods:**

Patients undergoing neurointerventional procedures were allocated to the sugammadex group (Group S) and the neostigmine group (Group N). Group S was administered IV 2 mg/kg sugammadex when a train-of-four (TOF) count of 2 was present, and Group N was administered neostigmine 50 mcg/kg with atropine 0.2 mg/kg at a TOF count of 2. We recorded heart rate, systolic blood pressure, diastolic blood pressure, mean blood pressure (MAP), and peripheral arterial oxygen saturation during administration of the reverse agent and at 2, 5, 10, 15, 30, 120 min, and 24 h thereafter. The primary outcome was blood pressure and heart rate change after the reversal agent was given. The secondary outcomes were systolic blood pressure variability standard deviation (a measure of the amount of variation or dispersion of a set of values), systolic blood pressure variability-successive variation (square root of the average squared difference between successive blood pressure measurements), nicardipine use, time-to-TOF ratio ≥0.9 after the administration of reversal agent, and time from the administration of the reversal agent to tracheal extubation.

**Results:**

A total of 31 patients were randomized to sugammadex, and 30 patients were randomized to neostigmine. Except for anesthesia time, there were no significant differences in any of the clinical characteristics between the two groups. The results demonstrated that the increase in MAP from period A to B was significantly greater in Group N than in Group S (regression coefficient = −10, 95% confidence interval = −17.3 to −2.7, *P* = 0.007). The MAP level was significantly increased from period A to B in the neostigmine group (95.1 vs. 102.4 mm Hg, *P* = 0.015), but it was not altered in Group S. In contrast, the change in HR from periods A to B was not significantly different between groups.

**Conclusion:**

We suggest that sugammadex is a better option than neostigmine in interventional neuroradiological procedures due to the shorter extubation time and more stable hemodynamic change during emergence.

## Introduction

The prevalence of intracranial saccular aneurysms is estimated to be 3.2% by imaging and autopsy series (1–3). Most intracranial aneurysms are asymptomatic throughout the life of patients. Nevertheless, subarachnoid hemorrhage, the most disastrous complication of aneurysm rupture, is associated with 50% mortality and 30–50% neurological morbidity in survivors (4). Endovascular coiling treatment of ruptured intracranial aneurysms reduces the absolute relative risk of death or dependence at 1 year by 7.4% (5).

Neuroradiological techniques have significantly improved the diagnosis and treatment of disease in the last decade (6). For better image quality because of patient immobilization and a more pleasant experience, general anesthesia is mostly performed rather than monitored anesthesia with neuroradiological techniques. However, there are some disadvantages to general anesthesia. First, the neurological examination cannot be assessed during the intraoperative period. In addition, intracranial pressure or blood pressure may increase due to endotracheal extubation or intubation. Regarding the possibility of aneurysm rupture due to acute elevation of blood pressure, the anesthetist needs to be able to monitor closely and exert control.

A neuromuscular blocking agent that keeps the patient immobilized during the surgery and facilitates endotracheal intubation is now integrated into the basic approach to anesthesia (7). Neostigmine is widely used to reverse neuromuscular blockade, inhibiting acetylcholinesterase and increasing the concentration of acetylcholine to counter non-depolarizing neuromuscular blockers at neuromuscular junction receptors. Nevertheless, neostigmine may increase the risk of arrhythmias, such as junctional rhythm, bradycardia, non-specific ECG changes, nodular rhythm, A-V block, or even asystole. To reduce its side effects, anticholinergic drugs such as atropine are used, but atropine may also induce the unwanted effects of tachycardia and arrhythmia (8–10). Sugammadex, a modified γ-cyclodextrin, has a high affinity for steroidal non-depolarizing neuromuscular blocking agents, which can expeditiously and totally reverse rocuronium's muscle relaxant effects. Because of its mechanism of action, sugammadex is thought to provide a faster and more predictable reversal of block. In addition, it can also avoid the unwanted side effects of neostigmine and antimuscarinic drugs (11–13). Sugammadex also does not affect heart rate, blood pressure, respiration, or thermoregulation in healthy patients (14).

Studies comparing sugammadex and traditional cholinesterase inhibitors with anticholinergic hemodynamic effects in neuroradiological techniques are limited. In a previous study, they compared sugammadex vs. neostigmine in patients having catheter-based neurointerventional procedures but they focused on extubation time and diaphragm recovery function but no cardiovascular response was studied (15). In our study, we aimed to compare the hemodynamic effects of sugammadex and neostigmine with atropine in neurointerventional procedures.

## Materials and methods

### Participants and study design

Our research protocol was approved by the Institutional Review Board of Chang Gung Memorial Hospital (Number: 202100679A3), and informed consent was obtained from patients. The trial was registered at ClinicalTrial.gov (NCT04997759). We included 61 patients who were scheduled for elective neurointerventional procedures at Chang Gung Memorial Hospital from September 2021 to April 2022. Patients who did not give written consent or those who were <20 years old, allergic to neuromuscular blocking drugs, difficult to intubate, experiencing end-stage renal disease, or pregnant were excluded.

In our randomized controlled study, we used a computer-generated randomization list, and the patients were randomly allocated into one of two groups, assigned to the sugammadex group (Group S) and the neostigmine group (Group N) at a ratio of 1:1. The patients were blinded for treatment.

### Anesthesia and tracheal extubation procedure

Propofol (2 mg/kg), rocuronium (1 mg/kg), and fentanyl (1 mcg/kg) were administered in induction. Anesthesia maintenance with sevoflurane with 100% O_2_ was performed using the anesthesia workstation (GE Avance Anesthesia Delivery System). We monitored muscle relaxation by a peripheral nerve stimulator (Datex-Ohmeda's M-NMT MechanoSensor™ and M-NMT ElectroSensor™), which was applied to the adductor pollicis using a train-of-four (TOF) mode, and we kept TOF counts of 0–1 during anesthesia. TOF was assessed until the ratio was ≥0.9 with a current of 70 mA. At the end of the surgery, patients in Group S were administered intravenous (IV) 2 mg/kg sugammadex when TOF count 2 was present, and Group N was administered neostigmine 50 mcg/kg with atropine 0.2 mg/kg. We recorded heart rate (HR), systolic blood pressure, diastolic blood pressure, mean blood pressure (MAP), and peripheral arterial oxygen saturation during administration of the reverse agent and at 2, 5, 10, 15, 30, 120 min, and 24 h thereafter. We removed the endotracheal tube when patients woke and reached a TOF ratio of ≥0.9. In addition, nicardipine was given when systolic blood pressure was >180 mmHg or diastolic blood pressure was >110 mmHg.

### Observational indices

In this study, we correct demographic data including age, sex, body weight, body height, comorbidity, and anesthesia time. Anesthesia time was defined as the administration of induction agents and ends with endotracheal extubation. TOF reach count 2 after induction was defined as the first time T2. The primary outcome was blood pressure and heart rate change after administration of the reversal agent. The secondary outcomes were (1) systolic blood pressure (SBP) variability standard deviation (a measure of the amount of variation or dispersion of a set of values); (2) systolic blood pressure variability-successive variation (square root of the average squared difference between successive blood pressure measurements); (3) nicardipine use; (4) time-to-TOF ratio ≥0.9 after the administration of the reversal agent (start from the time when the muscle relaxant reversal agent was administered and ends when TOF ratio ≥0.9); and (5) time from the administration of the reversal agent to tracheal extubation (start from the time when the muscle relaxant reversal agent was administered and ends with endotracheal extubation).

### Sample size

Sample size calculation was according to a previous study (16), which compared neostigmine to sugammadex in patients with neuromuscular blockade. The RR intervals at baseline and 10 min after reversal were 889 ± 106 ms and 849 ± 151 ms in the sugammadex group. The RR intervals at baseline and 10 min after reversal were 884 ± 122 ms and 915 ± 150 ms in the neostigmine group. Based on the reported data, the required minimum sample for both groups was 30, given the type I error of 5% and power of 80%.

### Statistical analysis

The clinical characteristics and secondary outcomes (e.g., extubation time) of patients receiving sugammadex vs. neostigmine were compared using Fisher's exact test for categorical variables or independent sample *t*-test for continuous variables. The change in vital signs (MAP and HR) from period A (averaging from reversal and both 2 and 5 min) to period B (averaging from 10 and 15 min) between groups was tested using a generalized estimating equation (GEE). The GEE model included intercept, main effects of study groups (sugammadex vs. neostigmine) and period (A vs. B), and an interaction term between group and period. The difference in the change value between groups was warranted once the interaction effect was statistically significant. The group difference at either period and the period difference (A vs. B) at either group were also investigated using the simple contrast within the GEE model. The link function was identity, and the distribution was normal in the GEE model. A two-sided *P*-value of <0.05 was considered to be statistically significant. Data analyses were conducted using SPSS 26 (IBM SPSS Inc., Chicago, Illinois).

## Results

A total of 61 patients were enrolled, of whom 31 and 30 patients were allocated to the sugammadex and neostigmine groups, respectively (Table 1). A total of 26 (43%) patients were men. The mean age was 56.2 ± 15.7 years. Most of the patients (93%) had an ASA classification of 3. Half of the patients (49%) had cardiovascular diseases. There were no significant differences in any of the clinical characteristics between the two groups except for the anesthesia time. The results showed that the anesthesia time was significantly shorter in the sugammadex group than in the neostigmine group (155.7 vs. 186.6 min, *P* = 0.037).

**Table 1:** Clinical characteristics of patients in the sugammadex and neostigmine groups.

**Variable**	**Total (*n* = 61)**	**Sugammadex (*n* = 31)**	**Neostigmine (*n* = 30)**	***P***
Male, Sex	26 (42.6)	14 (45.2)	12 (40.0)	0.797
Age, years	56.2 ± 15.7	58.5 ± 16.7	53.8 ± 14.5	0.241
Body height, m	1.62 ± 0.09	1.62 ± 0.09	1.62 ± 0.09	0.919
Body weight, kg	63.6 ± 11.1	63.1 ± 10.9	64.0 ± 11.5	0.754
Body mass index, kg/m^2^	24.23 ± 3.35	24.14 ± 3.61	24.33 ± 3.12	0.828
ASA classification				1.000
2	3 (4.9)	2 (6.5)	1 (3.3)	
3	57 (93.4)	29 (93.5)	28 (93.3)	
4	1 (1.6)	0 (0.0)	1 (3.3)	
Underlying disease				
Cardiovascular	30 (49.2)	16 (51.6)	14 (46.7)	0.800
Diabetes	8 (13.1)	3 (9.7)	5 (16.7)	0.473
Respiratory	2 (3.3)	1 (3.2)	1 (3.3)	1.000
Liver	4 (6.6)	2 (6.5)	2 (6.7)	1.000
Renal	2 (3.3)	0 (0.0)	2 (6.7)	0.238
Malignant	7 (11.5)	4 (12.9)	3 (10.0)	1.000
Induction vital signs				
HR, beats/ minute	75.39 ± 13.47	75.10 ± 13.13	75.70 ± 14.02	0.863
SBP, mm Hg	142.49 ± 19.39	142.39 ± 20.70	142.60 ± 18.29	0.966
DBP, mm Hg	80.9 ± 13.0	78.3 ± 10.8	83.7 ± 14.7	0.108
MAP, mm Hg	95.3 ± 12.3	93.4 ± 11.3	97.2 ± 13.2	0.225
SpO_2_, %	98.97 ± 1.37	98.81 ± 1.49	99.13 ± 1.22	0.354
Anesthesia time, minute	170.9 ± 58.3	155.7 ± 40.5	186.6 ± 69.5	0.037
First time T2, minute	67.7 ± 20.5	69.8 ± 17.0	65.6 ± 23.7	0.423

ASA, American Society of Anesthesiologists; HR, heart rate; SBP, systolic blood pressure; DBP, diastolic blood pressure; MAP, mean arterial pressure.

Data were presented as frequency (percentage) or mean ± standard deviation.

**Figure 1:**
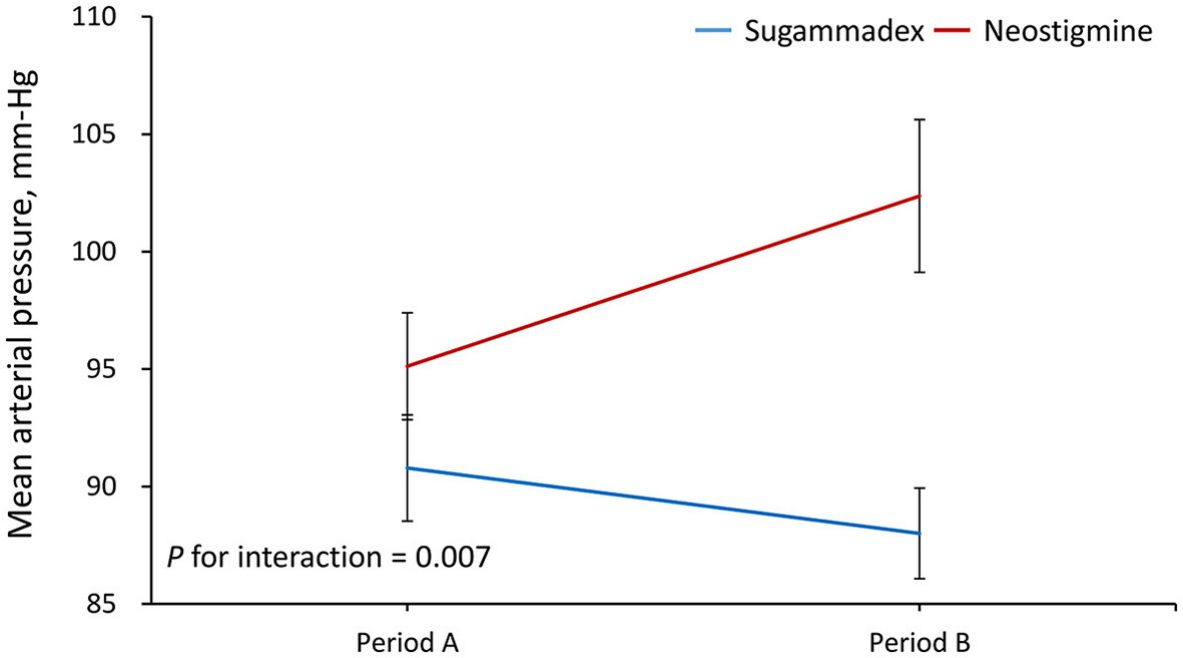
Changes in mean arterial pressure from period A to B. Period A was defined as the average from reversal, and both 2 and 5 min after reversal. Period B was defined as the average from 10 to 15 min after reversal.

**Figure 2:**
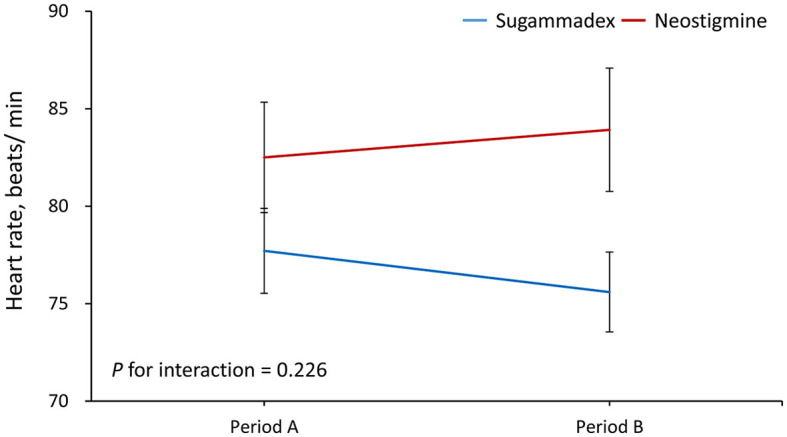
Changes in heart rate from period A to B. Period A was defined as the average from reversal, and both 2 and 5 min after reversal. Period B was defined as the average from 10 to 15 min after reversal.

Period A data was defined as the average from reversal, both 2 min and 5 min after reversal. Period B data was defined as the average from 10 min and 15 min after reversal. The results demonstrated that the increase in MAP from period A to B was significantly greater in Group N than in Group S (regression coefficient = −10, 95% confidence interval = −17.3 to −2.7, *P* = 0.007) (Figure 1). In addition, the MAP level was significantly increased from period A to B in Group N (95.1 vs. 102.4 mm Hg, *P* = 0.015), but it was not altered in Group S (Table 2). In contrast, the results showed that the change in HR from period A to B was not significantly different between groups (P for interaction = 0.226) (Figure 2; Table 2). As shown in the Supplementary Table, the mean arterial pressure also showed a significant difference 10 min after the reversal drug was administered.

**Table 2:** Comparison of vital signs between the sugammadex and neostigmine groups^*^.

	**Mean** ±**standard deviation**			
**Vital sign/Period**	**Sugammadex (S) (*****n*** = **31)**	**Neostigmine (N) (*****n*** = **30)**	***P-*** **value (S vs. N)**	***P*** **for interaction**
Mean arterial pressure, mm Hg				0.007
Period A (Reversal/2/5 min)	90.8 ± 12.6	95.1 ± 12.5	0.271	
Period B (10/15 min)	88.0 ± 10.7	102.4 ± 17.8	< 0.001	
*P-*value (period A vs. B)	0.212	0.015		
Heart rate, beats/minute				0.226
Period A (Reversal/2/5 min)	77.7 ± 12.1	82.5 ± 15.5	0.162	
Period B (10/ 15 min)	75.6 ± 11.4	83.9 ± 17.3	0.026	
*P-*value (period A vs. B)	0.310	0.488		

* The analysis was adjusted for anesthesia time.

The secondary outcomes between groups were also compared (Table 3). The results showed that compared to patients receiving neostigmine, those receiving sugammadex had a significantly smaller successive variation and standard deviation of SBP, were less likely to receive nicardipine, and had a shorter extubation time and the train-of-four ratio of 0.9 times (*P* < 0.05).

**Table 3:** The secondary outcome of patients in the sugammadex and neostigmine groups.

**Variable**	**Total (*n* = 61)**	**Sugammadex (*n* = 31)**	**Neostigmine (*n* = 30)**	***P***
SBPV SV, mm Hg	19.7 ± 7.7	16.4 ± 6.0	23.1 ± 7.8	< 0.001
SBPV SD, mm Hg	16.7 ± 6.1	14.5 ± 5.3	19.1 ± 6.1	0.003
Nicardipine				0.002
No	47 (77.0)	29 (93.5)	18 (60.0)	
Yes	14 (23.0)	2 (6.5)	12 (40.0)	
Extubation, seconds	617.3 ± 371.3	323.6 ± 136.9	920.9 ± 279.7	< 0.001
TOF ratio 0.9, s	554.1 ± 384.3	238.5 ± 116.5	880.3 ± 273.6	< 0.001

SBP, systolic blood pressure; SV, successive variation; SD, standard deviation; TOF, train-of-four.

Data were presented as frequency (percentage) or mean ± standard deviation.

$SV=\sqrt{1/\left(n-1\right)\overset{\left(n-1\right)}{\underset{\left(i=1\right)}{\sum }}{\left(BP_{i+1}-BP_{i}\right)}^{2}}$ and $SD=\sqrt{1/\left(n-1\right)\overset{\left(n-1\right)}{\underset{\left(i=1\right)}{\sum }}{\left(BP_{i}-BP_{mean}\right)}^{2}}$.

## Discussion

In this study, we evaluated the effect of sugammadex and neostigmine, both being neuromuscular blockade reversal agents, on hemodynamic changes during neurointerventional procedures. We found that sugammadex caused more stable hemodynamic changes and that the related parameter increases were more notable in patients who were administered neostigmine.

Compared with the conventional acetylcholinesterase inhibitor neostigmine, sugammadex permanently inactivates neuromuscular blocking agents, and it can reverse neuromuscular blockade of any depth because of its binding to rocuronium or vecuronium by 1:1. Due to its unique effects, anticholinergic drugs can be spared without any effect on the muscarinic receptor or plasma cholinesterase. For those with cardiovascular or respiratory disease, a lack of muscarinic and cardiovascular effects during emergence will be a significant benefit (17, 18).

During interventional neuroradiological procedures, reduced systolic blood pressure-successive variation was significantly associated with better functional recovery (19, 20). However, during emergence from anesthesia and endotracheal tube extubation, patients' vital signs fluctuated. Mild blood pressure elevation due to pain or excitement is often noted when recovering from anesthesia (21). Increased pulse rate and blood pressure were also seen in the extubation period because of the afferent pulse from the larynx that causes sympathetic activation (22). Tachycardia and hypertension with HR and BP elevations over 20% were noted at those times (23, 24). Stabilizing vital signs is critical for avoiding complications, particularly in patients with underlying cardiac or cerebrovascular disease. Patients who have poorly controlled hypertension may experience higher blood pressure than expected during emergence and extubation compared to normotensive patients, and the risk of myocardial ischemia, heart failure, pulmonary edema, or hemorrhagic stroke is elevated in such patients (23, 25).

In this study, we estimated the respective effect of the use of the reversal agent on patients' HR and MAP during two different time periods. The reversal agent was performed before the emergence from anesthesia. During period A (0–5 min), mean values of HR and MAP at 0 min (the time when the reversal agent was administered), 2 min, and 5 min after the use of the reversal agent were calculated. During period B (6–15 min), mean values of HR and MAP at 10 and 15 min after the administration of the reversal agent were evaluated. Period A (0–5 min) corresponded to the time when patients in both the neostigmine and sugammadex groups were intubated, and period B (6–15 min) represented the time when patients in the sugammadex group were extubated, while those in the neostigmine group were still intubated. We found that Group S patients remained stable in terms of HR or MAP during the whole emergence time during and after reversal compared with those patients in Group N, whose MAP and HR values rose remarkably in the first 15 min after reversal drugs were administered.

For MAP change, there was a major difference in period B between the N and S groups as well as between the N group in period A and the N group in period B, but there was no significant difference in the sugammadex group during period A or B. For HR change, there was also a major difference in period B between both the N and S groups. However, the change in HR from period A to B was not significant. We postulate that atropine may have influenced this consequence by rapidly affecting the heart rate increase within a few minutes. In contrast, sugammadex rapidly and completely reversed any effects that could cause slight changes, giving results that were in agreement with those of Sacan et al. (26).

Khuenl-Brady et al. (27) also showed that higher HR and blood pressure were noted in patients using neostigmine in a study of ASA I to III patients older than 18 years. In a randomized study by Lemmens et al. (12), 82 ASA I to IV patients were included, and they were administered sugammadex or neostigmine to reverse vecuronium under sevoflurane anesthesia. Increased HR from the baseline in the neostigmine group was noted compared to the sugammadex group. Hemodynamic stability in interventional neuroradiological procedures is important to prevent complications; however, related research is lacking. In our study, HR, MAP, and systolic and diastolic blood pressures were all higher in Group N after administration of the reversal agent, and the outcomes were similar to those of the above study.

Anti-hypertensive medication (nicardipine) was administered if patients had systolic blood pressure >180 mmHg or diastolic blood pressure >110 mmHg during the emergence period (28). In total, 12 patients in the neostigmine group required further control of their blood pressure in contrast to two patients in the sugammadex group; the result is consistent with our postulate that sugammadex has more stable hemodynamic control. Systolic blood pressure variation, successive variation, and standard deviation were also smaller in the sugammadex group. The median time to TOF reaching 90% after the reversal agent translated to 3.7-fold faster in the sugammadex group than in the neostigmine group. Our results are generally consistent with those of Sorgenfrei et al. (29), who reported that sugammadex reversed the neuromuscular block-to-TOF ratio by 90% within 5 min. In a Cochrane review by Hristovska et al. (30), it was also concluded that sugammadex (2 mg/kg) was 6.6 times faster than neostigmine (0.05 mg/kg) in reversing TOF count of 2 to TOF ratio 0.9.

Anesthesia time was significantly shorter in the sugammadex group, which is due to the shorter extubation time. A shorter extubation time with more stable hemodynamic change during emergence suggests that sugammadex is a better option than neostigmine in interventional neuroradiological procedures. It is notable that postoperative recurarization with a rapid increase in neuromuscular blockade after a period of recovery was not noted in either group after neuromuscular block reversal during extubation or in the postanesthesia care unit.

The advantages of sugammadex are not limited to neurointerventional procedures. Sugammadex is also beneficial for helping patients with brain injuries when the accurate neurologic examination is needed. The rocuronium-induced neuromuscular blockade for endotracheal intubation may cause muscle weakness, and sugammadex can rapidly reverse paralysis (31). A recent clinical study also showed that sugammadex played an important role in helping patients who underwent aortic valve replacement to improve postoperative recovery in cognitive domains (32).

There were several limitations in the present study. First, we focused on cardiovascular response during recovery from general anesthesia; thus, data on respiratory recovery only recorded peripheral arterial oxygen saturation, and further study is needed. Second, neither intra-rater nor inter-rater reliability assessments were conducted, and therefore, the results might be influenced by the potential measurement error.

In conclusion, this study demonstrates that sugammadex provides more hemodynamic stability and expeditiously reverses moderately deep rocuronium-induced neuromuscular blockade without unpredictable side effects. Furthermore, these preliminary data also suggest superiority over the widely used anti-cholinesterase due to the greater comprehensiveness and speed of the reversal process. We suggest that using sugammadex is more advantageous than neostigmine in interventional neuroradiological procedures.

## Data availability statement

The original contributions presented in the study are included in the article/Supplementary material, further inquiries can be directed to the corresponding author.

## Ethics statement

The studies involving human participants were reviewed and approved by the Institutional Review Board (IRB) of Chang Gung Memorial Hospital (Number: 202100679A3). Written informed consent to participate in this study was provided by the patients/participants or patients/participants' legal guardian/next of kin.

## Author contributions

All authors listed have made a substantial, direct, and intellectual contribution to the work and approved it for publication.
