# Supplementary material for: Comparison of neostigmine and sugammadex for hemodynamic parameters in neurointerventional anesthesia
Source: Front Neurol. 2023 Apr 17;14:1045847. doi: 10.3389/fneur.2023.1045847 (PMC10150384; doi:10.3389/fneur.2023.1045847)
Supplement: Supplementary file 1 [file Data_Sheet_1.PDF]

Supplemental Table 1. The comparison of vital signs between the sugammadex and neostigmine groups*

Vital sign/ Time	Sugammadex (S) (*n* = 31)	Neostigmine (N) (*n* = 30)	*P* of group within time	*P* of S (vs. Reversal)	*P* of N (vs. Reversal)
Mean arterial pressure, mm Hg					
Reversal	84.5 ± 14.6	87.6 ± 14.6	1.000	-	-
2 minute	95.5 ± 18.3	98.8 ± 15.6	1.000	0.058	0.031
5 minute	92.4 ± 16.3	99.0 ± 20.0	1.000	1.000	0.259
10 minute	88.9 ± 13.2	105.3 ± 22.0	0.040	1.000	<0.001
15 minute	87.1 ± 10.7	99.4 ± 20.3	0.326	1.000	0.074
30 minute	92.6 ± 10.1	93.4 ± 10.1	1.000	0.594	1.000
2 hour	95.2 ± 11.3	98.9 ± 13.5	1.000	0.062	0.001
24 hour	90.5 ± 10.2	89.8 ± 12.7	1.000	1.000	1.000
Heart rate, beats/ minute					
Reversal	74.5 ± 14.4	76.3 ± 17.4	1.000	-	-
2 minute	80.5 ± 15.6	88.9 ± 15.6	1.000	0.555	<0.001
5 minute	78.1 ± 13.7	82.3 ± 19.5	1.000	1.000	1.000
10 minute	76.3 ± 12.5	81.9 ± 20.1	1.000	1.000	1.000
15 minute	74.9 ± 12.2	85.9 ± 18.4	0.599	1.000	0.071
30 minute	70.8 ± 12.7	76.6 ± 18.4	1.000	1.000	1.000
2 hour	69.7 ± 12.4	70.9 ± 9.8	1.000	1.000	1.000
24 hour	76.9 ± 11.4	78.8 ± 12.7	1.000	1.000	1.000

* The analysis was adjusted for anesthesia time;

Data were presented as mean ± standard deviation.
